# New Models of Microsporidiosis: Infections in Zebrafish, *C. elegans*, and Honey Bee

**DOI:** 10.1371/journal.ppat.1001243

**Published:** 2011-02-17

**Authors:** Emily R. Troemel

**Affiliations:** Section of Cell and Developmental Biology, University of California San Diego, La Jolla, California, United States of America; University of California San Francisco, United States of America

## Microsporidia Are Minimalist Parasites That Invade and Replicate inside a Wide Range of Hosts

**Figure 1:**
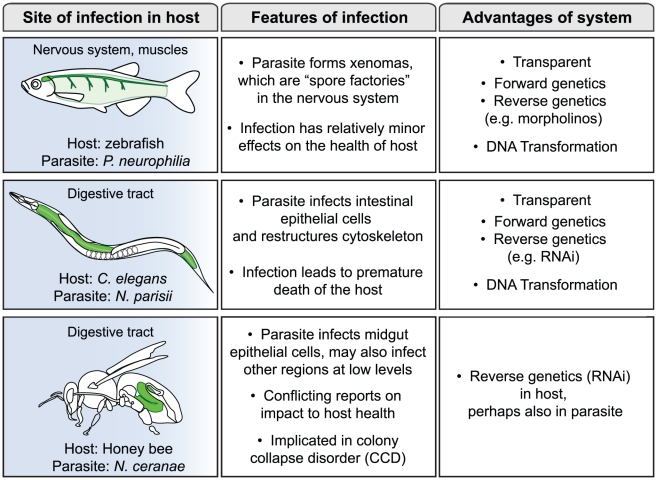
Three new systems for study of infection by microsporidia. Zebrafish, *C. elegans*, and honey bee are infected by distinct species of microsporidia. Site of infection is highlighted in green for each host. Transmission in each system likely occurs via a fecal-oral route and the host is infected by ingesting infectious spores. Diagram by Malina Bakowski.

Microsporidia comprise a large phylum of fungal-related pathogens that have been studied since the time of Louis Pasteur, who in 1870 found that they were responsible for silkworm disease that was decimating the silkworm industry [1], [2]. These obligate intracellular microbes are ubiquitous, but have remained enigmatic because of the difficulties of culturing them in the lab. In the 1990s there was a surge of interest in microsporidia when it was found that they were responsible for severe diarrhea and death in AIDS patients, but most research on these parasites has been conducted in fish and insects [3]. A recent *PLoS Pathogens* Pearl focused on the phylogenetic placement of microsporidia and the compactness of their genomes [4]. In this review we will consider the interaction between microsporidia and their hosts, with a focus on three non-mammalian hosts: zebrafish, *Caenorhabditis elegans*, and honey bee (Figure 1). These hosts are relatively new systems for the study of microsporidia, with distinct reasons motivating interest in each of them as described below. These systems provide exciting new opportunities to obtain insights into the mechanisms of microsporidia pathogenesis.

Microsporidia as a group are able to infect an astonishingly wide range of hosts, including all animal phyla as well as a few protists. But how specific is the host range for a particular species of microsporidia? Roughly 1,200 species of microsporidia have been described, and it is difficult to make a blanket statement about their host range. On one end of the spectrum is *Antonospora locustae*, which appears to have a very narrow host range. *A. locustae* infects and eventually kills locusts, and has been approved by the United States Environmental Protection Agency as a “green pesticide” for locust control. In the process of obtaining approval for agricultural use, it was shown that *A. locustae* did not infect mammals, birds, fish, aquatic invertebrates, or honey bees (http://www.epa.gov/). On the other end of the spectrum is *Anncallia* (formerly *Brachiola* or *Nosema*) *algerae*, which was originally isolated from mosquitoes, but can replicate in several different species of invertebrate and vertebrate cell lines [5]. Interestingly, *A. algerae* appears to have caused death in an immunocompromised patient, possibly from a mosquito bite [6]. Thus microsporidia may have narrow or wide host range, depending on the species. The determinants of this host range are likely to encompass both host and pathogen factors that allow microsporidia infection.

How do microsporidia infect their hosts? Many microsporidia use a fecal-oral route of transmission, with some species restricted to intestinal cells and other species disseminating to a variety of tissues and organs. Very generally, microsporidia have two life stages: the actively replicating meront that develops inside of host cells, and the dormant spore form, which is the transmissible form that survives outside the host cell. In order to invade cells, spores contain a highly specialized infection apparatus called a polar tube. This tube is coiled inside the spore and then “fires” outside the spore to directly inject the parasite into host cells, although the exact details of this process are poorly understood in vivo. A variety of cues have been shown to induce polar tube firing in vitro, but no cue has been found that is universal [7]. Presumably the host environment encountered by each microsporidia species during infection differs, and thus each species may respond to a distinct set of conditions.

Once inside the host cell, microsporidia are very dependent occupants. Microsporidian genomes are extremely reduced in size, having jettisoned functions that can be accomplished by the host cell. An example of this dependence is that microsporidia cannot make ATP (except perhaps through substrate level phosphorylation) and instead appear to steal this vital energy currency from the host. This strategy of dramatic invasion followed by extreme dependence on the host cell appears to be evolutionarily successful, since there are a large number of species and hosts for microsporidia. A lifestyle such as this may represent the “bare bones” of a eukaryotic pathogen and the closest lifestyle to a virus of any eukaryote. Thus, study of microsporidia may provide insight into what represents the minimal arsenal for a eukaryotic parasite.

## The Zebrafish *Danio rerio* Is a Host for Microsporidia

About 100 species of microsporidia have been shown to infect fish, including agriculturally relevant fish, such as salmon and rainbow smelt [8]. Indeed, the decline of entire fisheries has been attributed to microsporidiosis on several occasions. With the advent of zebrafish as a genetic model system, the infection of zebrafish by microsporidia has been increasingly observed. In fact, analyses from the University of Oregon diagnostic service indicated that microsporidia are the most common cause of disease for laboratory zebrafish [9], making this pathogen a serious concern for zebrafish researchers. The most commonly observed species that infects zebrafish is *Pseudoloma neurophilia* [10]. As is often the case in fish microsporidia infections, *P. neurophilia* form complexes called xenomas, which are essentially spore factories that generate vast quantities of spores. *P. neurophilia* xenomas are found in the nervous system, such as the hind brain, spinal cord, nerve roots, and occasionally in muscle (Figure 1). Despite the substantial size and frequency of these xenomas, they appear to be a relatively minor burden on the health and lifespan of the fish. Therefore, in this system microsporidia appear well-adapted to exploit their hosts by maximizing spore production but minimizing impact on the host.

## The Nematode *C. elegans* Is a Host for Microsporidia

The nematode *C. elegans* is another genetic model organism that has recently been a focus for the study of microsporidia infections. *C. elegans* has been an extremely useful system for addressing many biological questions since 1970, including host defense and pathogenesis more recently [11]. However, most studies in the field of *C. elegans* pathogenesis have involved clinically relevant human pathogens that were not known to be natural pathogens of this animal. In a search for natural pathogens of *C. elegans*, a new genus and species of microsporidia was found in a wild-caught strain of *C. elegans* isolated from a compost pit near Paris [12]. This new species was named *Nematocida parisii*, or nematode-killer from Paris. In addition to this species, several other wild-caught *Caenorhabditis* nematodes have been isolated that harbor microsporidia ([12]; M-A. Félix, personal communication). Infection with *N. parisii* eventually leads to premature death of the host, but nematodes can carry a substantial parasite burden and still feed and move relatively normally for some time. One interesting aspect of the infection is that *N. parisii* appears to restructure the cytoskeleton of *C. elegans* host cells, perhaps as part of a non-damaging exit strategy (Figure 1). This restructuring may again be an example of microsporidia maximizing spore production and transmission, but minimizing impact on the host.

## The Honey Bee *Apis mellifera* Is a Host for Microsporidia

In addition to the two genetic model hosts described above, microsporidia are also common in agriculturally relevant organisms, such as *Apis mellifera*, the Western honey bee. Precipitous drops in honey bee numbers have been observed in recent years, a phenomenon referred to as honey bee colony collapse disorder (CCD) [13]. Because honey bees are responsible for pollinating crops of economic importance, such as almonds, berries, fruits, and vegetables, this die-off is of great concern. The reason for this die-off is controversial, and some have even questioned whether it is significantly different from episodic declines in the past. In any case, there has been an active search for pathogens that could be responsible for CCD. The microsporidian species *Nosema apis* has long been known to afflict Western honey bee colonies, and in recent years, a new species of microsporidia called *Nosema ceranae* has increasingly been found in Western honey bee colonies around the world [14]. Study of *N. ceranae* has been an active area of interest as a possible cause of CCD. Some reports have indicated that *N. ceranae* is more pathogenic to honey bees than *N. apis* and could be a cause of CCD [15], although other studies have not found a difference in pathogenicity between the two species [16]. A recent report may provide a reconciliation of these conflicting results: this study indicates that CCD requires infection by both *N. ceranae* and a virus [17]. Clearly, *N. ceranae* warrants further study for its potential contribution to CCD, and for its general effects on the health of honey bee colonies.

## Why Do These Systems Provide Fertile Ground for Study, and What Can Be Learned from Them?

After microsporidia invade host cells they undergo elaborate development, which has been described in rich detail through decades of electron microscopy (EM) in a variety of host/parasite pairs. However, these studies lack kinetic information, since EM provides only a single snapshot and requires labor-intensive fixation, sectioning, and staining to visualize the infection. For this reason, the transparent hosts *C. elegans* and zebrafish provide excellent systems for analysis of microsporidia development, since infections can be visualized inside living, intact animals. *C. elegans* and zebrafish provide advantages over other hosts like mammals and insects, which require dissection to analyze infection, even for standard microscopy studies. Individual *C. elegans* and zebrafish animals can repeatedly be analyzed microscopically throughout infection, making it possible to track the kinetics of parasite development inside these transparent and hardy hosts.

Another limitation of previous microsporidia studies is the relative dearth of molecular information. The field of microsporidia pathogenesis is full of rich cell biology questions, which have been underexplored molecularly due to a lack of tools. In particular, how do microsporidia exploit and restructure their hosts in order to minimize their impact, such as for xenomas in the fish and for cytoskeletal restructuring in *C. elegans*? Do microsporidia secrete enzymes to perform this work, or do they intersect pathways further upstream and instruct the host to do its own restructuring? Again, answers to these questions may come from imaging and genetic studies in genetically accessible hosts such as *C. elegans* and zebrafish. Both the zebrafish and *C. elegans* communities have generated strains that express fluorescently tagged proteins to allow for analysis of host molecules in live animals. In our own studies we have used such strains to reveal dramatic changes in host cytoskeletal proteins during infection (unpublished observations). In addition, *C. elegans* and zebrafish provide powerful genetic tools that allow for unbiased identification of host proteins involved in microsporidia infection through forward genetic screens. Proteins identified in these studies can then be examined in a more directed manner for their roles in less tractable hosts such as mice and humans.

In contrast to the above systems, the honey bee has not traditionally been a strong genetic system, but reverse genetics have recently been developed in the honey bee with the use of RNA interference [18]. This technique should allow for directed investigations into the roles of honey bee proteins in microsporidia infection. A tissue culture system has also been developed for *N. ceranae* that will facilitate study [19]. Intriguingly, RNAi may also be possible in the parasite itself. The genome sequence of *N. ceranae* indicates that the RNAi pathway is intact in this parasite, and an exciting report suggests that *N. ceranae* genes can be silenced by feeding dsRNA to the honeybee host [20]. This may be the first example of genetic manipulation in microsporidia, and opens up enormous potential for doing functional analysis of microsporidian genes.

Analysis of other microsporidian species will be facilitated by the Microsporidian Genomes Consortium effort at the Broad Institute (http://www.broadinstitute.org/files/shared/genomebio/Microsporidia_wp.pdf). The genomes of several species are being sequenced, including *N. parisii* and *P. neurophilia*. Sequence data from these small genomes will allow for molecular analysis, perhaps through RNAi or misexpression studies in the host. Comparative studies such as these will likely yield insight into what endows each species with its own characteristics and ability to interact and exploit its specific host. They may also provide insight into the general strategies used by microsporidia, which are some of the most streamlined eukaryotic parasites.
